# Mutation of the second sialic acid-binding site of influenza A virus neuraminidase drives compensatory mutations in hemagglutinin

**DOI:** 10.1371/journal.ppat.1008816

**Published:** 2020-08-27

**Authors:** Wenjuan Du, Margreet A. Wolfert, Ben Peeters, Frank J. M. van Kuppeveld, Geert-Jan Boons, Erik de Vries, Cornelis A. M. de Haan

**Affiliations:** 1 Section of Virology, Division of Infectious Diseases & Immunology, Department of Biomolecular Health Sciences, Faculty of Veterinary Medicine, Utrecht University, Utrecht, The Netherlands; 2 Department of Chemical Biology and Drug Discovery, Utrecht Institute for Pharmaceutical Sciences, and Bijvoet Center for Biomolecular Research, Utrecht University, Utrecht, the Netherlands; 3 Complex Carbohydrate Research Center, University of Georgia, Athens, United States of America; 4 Wageningen Bioveterinary Research, Department of Virology, Lelystad, the Netherlands; University of Georgia, UNITED STATES

## Abstract

Influenza A viruses (IAVs) cause seasonal epidemics and occasional pandemics. Most pandemics occurred upon adaptation of avian IAVs to humans. This adaptation includes a hallmark receptor-binding specificity switch of hemagglutinin (HA) from avian-type α2,3- to human-type α2,6-linked sialic acids. Complementary changes of the receptor-destroying neuraminidase (NA) are considered to restore the precarious, but poorly described, HA-NA-receptor balance required for virus fitness. In comparison to the detailed functional description of adaptive mutations in HA, little is known about the functional consequences of mutations in NA in relation to their effect on the HA-NA balance and host tropism. An understudied feature of NA is the presence of a second sialic acid-binding site (2SBS) in avian IAVs and absence of a 2SBS in human IAVs, which affects NA catalytic activity. Here we demonstrate that mutation of the 2SBS of avian IAV H5N1 disturbs the HA-NA balance. Passaging of a 2SBS-negative H5N1 virus on MDCK cells selected for progeny with a restored HA-NA balance. These viruses obtained mutations in NA that restored a functional 2SBS and/or in HA that reduced binding of avian-type receptors. Importantly, a particular HA mutation also resulted in increased binding of human-type receptors. Phylogenetic analyses of avian IAVs show that also in the field, mutations in the 2SBS precede mutations in HA that reduce binding of avian-type receptors and increase binding of human-type receptors. Thus, 2SBS mutations in NA can drive acquisition of mutations in HA that not only restore the HA-NA balance, but may also confer increased zoonotic potential.

## Author summary

Influenza pandemics can have devastating effects as was dramatically demonstrated by the so-called Spanish flu in 1918. Such pandemics are caused by animal influenza A viruses (IAVs) that are sufficiently adapted to humans. This adaptation includes among others a change in the receptor-binding properties of the viral hemagglutinin (HA). Some avian IAVs have already acquired the ability to bind to human-type receptors via their HA. We provide novel insights into the evolution of such viruses and elucidate a crucial role for NA herein. This study is expected to contribute to the timely recognition of animal viruses with increased zoonotic potential.

## Introduction

Influenza A viruses (IAVs) cause seasonal epidemics as well as occasional pandemics. The latter occur when animal viruses cross the host species barrier and adapt to humans. IAV particles contain hemagglutinin (HA) and neuraminidase (NA) glycoproteins with receptor-binding and -cleavage activities, respectively. HA binds to glycans terminating in N-acetylneuraminic acid (Neu5Ac; generally known as sialic acid [SIA]) and is a major host tropism determinant. Human viruses prefer binding to α2,6-linked SIA (α2,6 SIA; human-type receptor) expressed on human upper airway epithelia whereas most avian viruses prefer binding to α2,3-linked SIA (α2,3 SIA; avian-type receptor) present on bird intestinal epithelia [1]. Some avian viruses, such as recent H7N9 and H9N2 viruses, however also can bind to α2,6 SIA [2, 3] and are therefore regarded as viruses with increased zoonotic potential. What drives the selection of mutations in HA resulting in the binding of human-type receptors in these avian viruses is, however, not understood.

As yet, little is known about the importance of the NA in host tropism changes. Although the NAs of human IAVs are relatively better at cleaving α2,6 SIA they still, like avian IAVs, display a higher cleavage rate cleavage of α2,3 SIAs [4–8]. It has been suggested that not HA binding per se, but rather the binding properties of HA in relation to the activity of NA are important for optimal virus replication, fitness and host tropism [9–12]. HA and NA likely require a functional balance that matches the host sialome in order to escape from (mucus) decoy receptors, to enable cell attachment and endocytic uptake, and to allow release of newly assembled virus particles at the end of the infection cycle [12]. Importantly, high variability in the receptor-binding properties of HA (avidity and receptor fine-specificity) for avian as well as human IAVs has been observed [13–15], which may need to be accompanied by compensatory mutations in NA to maintain a functional balance.

The NA protein is a type II transmembrane protein assembling into a mushroom-shaped homo-tetramer. Each globular head domain contains a catalytic site, which is highly conserved between IAVs of different subtypes [6, 7]. A grossly neglected feature of NA is a second sialic acid-binding site (2SBS; also referred to as hemadsorption site) [16–19] adjacent to the catalytic site. The 2SBS consists of three loops (370, 400 and 430 loop) that contain residues interacting with SIA [17, 18]. A functional 2SBS enhances NA activity on multivalent, but not monovalent, substrates, probably by bringing substrates closer to the active site [4, 5, 16]. The presence or absence of a functional 2SBS was shown to affect virus replication in vitro [4, 5, 20–22], presumably by affecting the often mentioned, but poorly characterized HA-NA balance [20]. Of note, the 2SBS is conserved in most avian viruses but is invariably lost in human viruses as well as in viruses from some other hosts [5, 16, 18, 20, 23]. This suggests an important, although so far unproven, role for the 2SBS in host tropism.

Recently, kinetic assays based on bio-layer interferometry (BLI) were introduced to study the balance between HA and NA in the context of virus particles. These assays allow monitoring of HA binding and NA cleavage in real time using synthetic glycans or sialylated glycoproteins [20, 24]. IAV particles are practically irreversibly attached to a receptor-coated surface, in the absence of NA activity, due to multivalent HA-receptor binding. The combined activity of HA and NA, however, results in virion movement on a receptor-coated surface until the receptor density is sufficiently decreased by receptor-destroying NA to allow virion release. The speed of this self-elution from a receptor-coated surface is determined by the avidity of HA and the activity of NA for a specific receptor, and thus reflects the HA-NA-receptor balance. By using BLI, N2-containing human viruses with a functional 2SBS elute faster from a receptor-coated surface than those without, indicating that the HA-NA balance can be affected by the receptor-binding properties of the 2SBS of NA in addition to those of HA [20, 24].

In the present study we show that mutation of the 2SBS in NA disturbs the HA-NA balance of avian H5N1 virus. Importantly, we establish the existence of a functional HA-NA crosstalk by showing that a mutated, non-functional 2SBS drives the selection of mutations in HA that modify its receptor-binding properties, thereby restoring the HA-NA balance. As a corollary, an acquired mutation also resulted in concomitant enhanced binding to human-type receptors. These findings are corroborated by phylogenetic analyses of H9N2 viruses. Also for H9N2 viruses mutations in the 2SBS are selected prior to the acquisition of mutations in the receptor-binding site (RBS) of HA that decrease binding to avian-type receptors and increase binding to human-type receptors. Collectively, these results emphasize the importance of the 2SBS for the HA-NA balance of avian viruses and provide an explanation for the evolution of avian viruses that bind to human-type receptors.

## Results

### Mutation of the 2SBS affects the HA-NA balance of H5N1 virus and selects for mutations in HA

**Fig 1:**
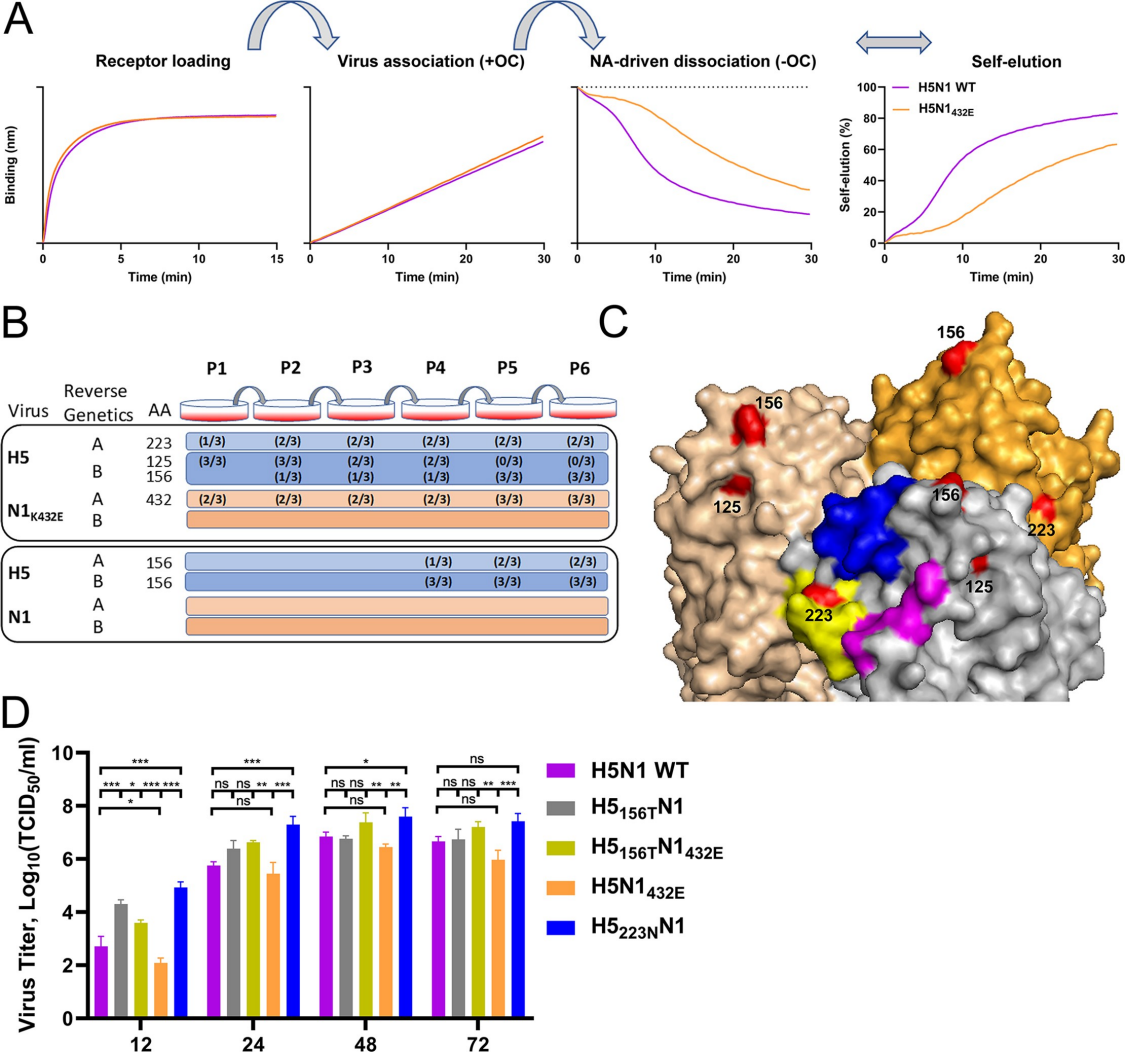
Mutation of the 2SBS affects the HA-NA balance of H5N1 virus and selects for mutations in HA. (A) Streptavidin-containing BLI sensors were loaded with biotinylated LAMP1, generating the receptor loading curve (Receptor loading). LAMP1 contains more α2,3 than α2,6 SIAs [20]. Subsequently, sensors were dipped into solution containing H5N1 or H5N1_432E_ viruses (for a list of the virus stocks used see S3 Table) and the NA inhibitor oseltamivir-carboxylate (OC), resulting in a virus association curve [Virus association (+OC)]. Viruses were loaded to a similar loading level. NA-driven virus dissociation was observed when OC was removed by 3 short (5 s) washes [NA-driven dissociation (-OC)]. A virion self-elution graph was generated by normalizing virus dissociation to the virus association level in the presence of OC (Self-elution). A representative experiment out of three performed is shown. (B) Overview of the HA and NA mutations that were detected upon passaging of H5N1_432E_ and H5N1 viruses. For an elaborate overview see S1 Table and S2 Table. Viruses were generated twice by reverse genetics (indicated by A and B), after which they were each passaged in triplicate. Amino acid (AA) positions of mutations in HA (H125N, A156S/T, S223N) and NA (E432K) are indicated. Numbers between brackets indicate the occurrence of these mutations in the triplicate passage series. Mutation S223N was always detected in combination with E432K, which restored the 2SBS. Mutation at position 125 was followed by mutation at position 156, after which the mutation at position 125 disappeared from the population. Mutation at position 156 was also observed after several passages in H5N1 virus. (C) Surface representation of the crystal structure of the H5 trimer from A/Vietnam/1194/04 (H5N1) (PDB ID: 2IBX; [29]) was depicted using Pymol software. Head domains are shown. The three structural elements of the RBS are colored in blue (190-helix), yellow (220-loop) and magenta (130-loop). Residues in HA that are mutated during passaging of H5N1 viruses are shown in red and numbered. (D) MDCK-II cells were infected (n = 3) with the indicated viruses (see S3 Table for details) at multiplicity of infection of 0.001 TCID_50_ units per cell. The virus titer in the cell culture supernatants at the indicated times post infection was determined by limiting dilution followed by calculation of the TCID_50_ titers. Standard deviations are indicated. Significant differences were analyzed by One-way ANOVA using Graphpad (*, P<0.05; **, P<0.01; ***, P<0.001; n.s., not significant).

We previously generated an H5N1 virus with mutation K432E in the 2SBS of NA (H5N1_432E_) [5] to analyze the importance of the 2SBS for NA catalytic activity and virus replication. This mutation, which reduced catalytic activity of NA on multivalent, but not monovalent substrates [5], resulting from reduced receptor binding via the 2SBS (S1 Fig), rapidly reverted back to wild type NA (432K)[5]. This observation prompted us to examine the effect of mutation K432E in NA on the HA-NA balance of H5N1 virus. To this end, we used a recently established BLI assay [20, 24](Fig 1A), in which sensors were coated with recombinant soluble lysosomal-associated membrane glycoprotein 1 (LAMP1) receptors that carry several sialoglycans (receptor loading). The sensors were subsequently loaded with virus particles in the presence of the NA inhibitor oseltamivir-carboxylate (OC) [Virus association (+OC)], after which we determined the rate of NA-activity-dependent dissociation in the absence of OC [NA-driven dissociation (-OC)]. The virus self-elution rate (NA-driven dissociation normalized to the virus association level) is a reflection of the HA-NA balance where, for instance, viruses containing HA with low receptor-binding affinity or NA with high catalytic activity are released faster than viruses that bind stronger and/or have less active NA proteins [24]. Comparison of wild type H5N1 (H5N1 WT) and H5N1_432E_ viruses carrying the same H5 protein, showed that mutation K432E in NA disturbs the HA-NA balance as it resulted in slower virion self-elution (Fig 1A).

**Fig 2:**
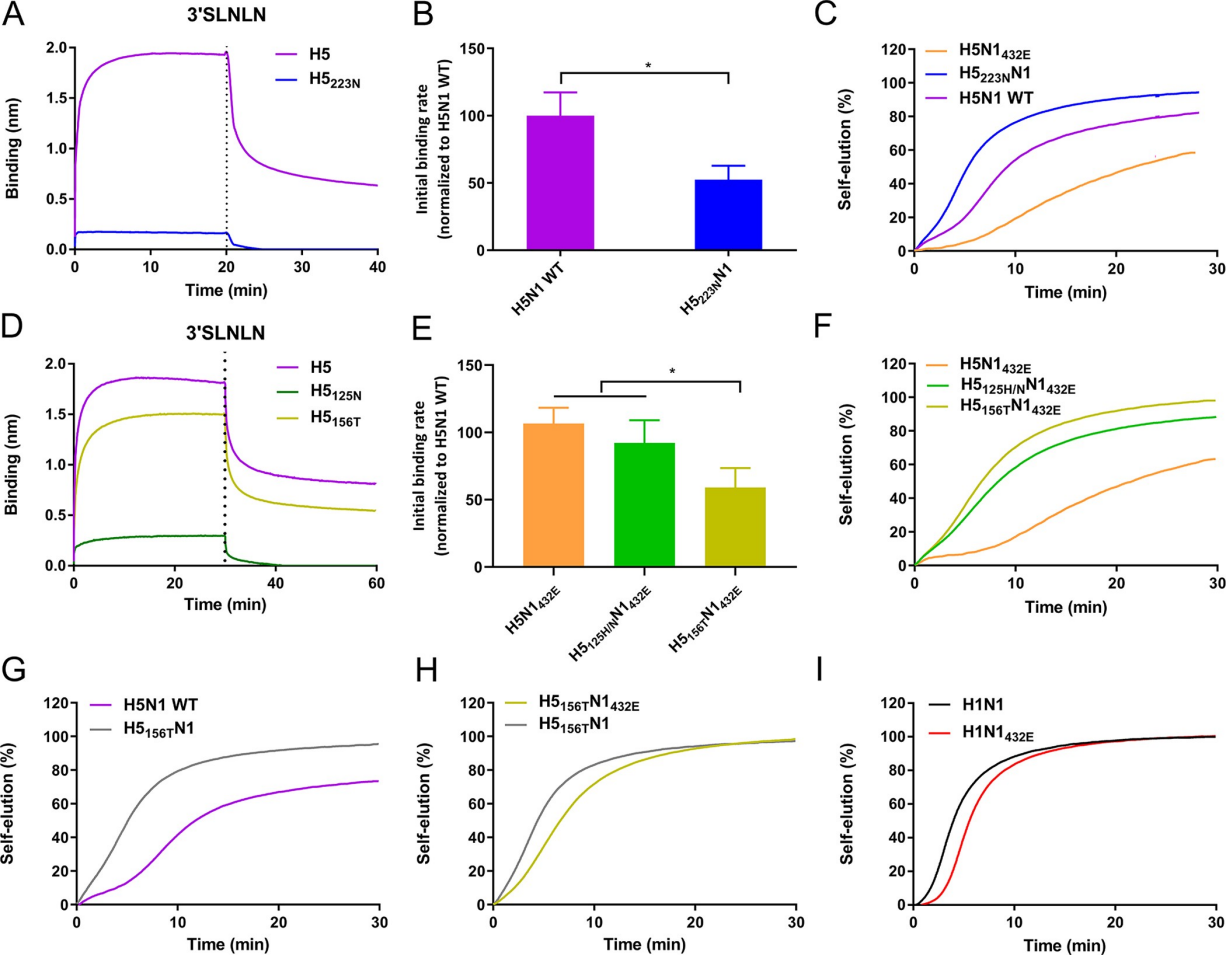
Mutations in HA restore the HA-NA balance. (A and D) Association and subsequent dissociation of indicated H5 proteins complexed with antibodies to and from 3’SLNLN-coated sensors. (B and E) Relative initial binding rates per virus particle (v_obs_ = dB/dT) for LAMP1-coated sensors were determined for H5N1 and H5N1 mutant viruses (H5_223N_N1, H5_156T_N1_432E_, H5_125H/N_N1_432E_, and H5N1_432E_) as described previously [20, 24] and graphed normalized to H5N1. Means of three independent experiments are graphed, standard deviations are shown. The relative initial binding rate per virus particle corresponds to the steepness of the initial part of binding curves (as show in Fig 1) normalized for virus particle numbers. (C and F) NA-driven self-elution of H5N1 and H5N1 mutant viruses from LAMP1-coated sensors was analyzed similarly as described in the legend to Fig 1. (G and H) NA-driven self-elution of H5N1 and H5N1 mutant viruses (H5_156T_N1 and H5_156T_N1_432E_) from LAMP1-coated sensors. (I) Self-elution of H1N1 and H1N1_432E_ viruses from LAMP1-coated sensors. Representative experiments out of 2–3 performed are shown.

In our previous work, revertant E432K was readily obtained upon passaging of H5N1_432E_ [5]. To restore the altered HA-NA balance, compensatory mutations in HA may also suffice to compensate for substitution K432E in NA. Therefore, recombinantly generated H5N1 WT and H5N1_432E_ viruses were serially passaged on MDCK cells. Each virus was generated twice by reverse genetics and passaged in triplicate series at low multiplicity of infection (MOI 0.001)(Fig 1B). After each passage, viral RNA was extracted and HA and NA genes were sequenced. For H5N1_432E_, back mutation E432K was observed in three out of the six passage series. In two series this was combined with mutation S223N in H5 (S227N according to H3 numbering) (S1 Table; Fig 2A). Mutation H125N in H5 (H129N according to H3 numbering) was observed in the other three series after only one passage (S1 Table; Fig 1B). Upon passaging, substitution H125N was, however, lost in all these series concomitantly with the appearance of A156T in H5 (A160T according to H3 numbering) (S1 Table; Fig 1B). Sequencing results of different passages of H5N1_432E_ are shown in S2 Fig. Much fewer mutations (requiring at least four passages) were obtained upon passaging of H5N1 WT (S2 Table, Fig 1B). For five passage series of H5N1 WT, substitution A156T was observed in H5, while in one series additional mutation A156S was detected. No mutations were observed in NA upon passaging of H5N1 WT (S2 Table).

Two of the observed HA mutations are located in (S223N; in the 220 loop) or close to (H125N; proximal of the 130 loop) the RBS of H5 (Fig 1C). Substitutions A156T and A156S, generating a N-linked glycosylation site (NXS/T; S3 Fig), are located at the tip of the head domain as confirmed by an increased electrophoretic mobility of recombinant soluble H5 proteins with substitution A156T (S4 Fig). In conclusion, mutation of the 2SBS in H5N1 rapidly selects for mutations in NA that restore the 2SBS and/or for mutations in HA located in or close to the RBS or resulting in the addition of a glycan chain to the HA head domain.

The selection of the mutations in HA indicates that viruses carrying these mutations are more fit and replicate more efficiently in MDCK cells. To analyze the effect of these HA mutations in more detail, we determined virus growth curves on MDCK cells. All mutant viruses reached higher titers than their respective parental viruses at the early time point (Fig 1D). Differences in viral titers were smaller at the later time points. Also the two parental viruses (H5N1 WT and H5N1_432E_) obtained significantly differed titers at the early time point, indicating that a disturbance of the HA-NA balance of H5N1 virus, resulting in slower virion self-elution as determined by BLI, negatively affects virus replication.

### Effect of compensatory mutations in HA on receptor binding and virion-self-elution

Mutations in the H5 head domain may affect receptor binding and therefore HA-NA balance. We expressed recombinant soluble H5 proteins carrying the different mutations (H5_223N_, H5_125N_, and H5_156T_) and analyzed their receptor binding properties. Each of the three mutations negatively affected the H5 protein binding rate to an avian-type synthetic glycan (3’SLNLN; NeuAcα2-3Galβ1-4GlcNAcβ1-3Galβ1-4GlcNAc) as observed using BLI (Fig 2A and 2D). The effect of the different mutations on receptor binding was also analyzed in the context of virus particles by using BLI, by analyzing the initial binding rate of virions to the sensor surface in the presence of NA inhibitor OC similarly as described previously [24]. In this analysis, virions carrying the same NA protein (either the wildtype or the 432E NA) were compared. Substitutions S223N and A156T resulted in significantly lower initial binding rates (Fig 2B and 2E). As virus stocks carrying 125N in the absence of wildtype 125H were not obtained (S2 Fig and S1 Table), we determined the initial binding rate of virus stocks containing both 125H and 125N in HA as determined by sequence analysis (H5_125H/N_N1_432E_). The initial binding rate observed for these latter virus stocks did not differ compared to H5N1_432E_ (Fig 2E). In short, mutations at position 223 and 156 in H5 result in reduced HA-receptor binding in both assays used. The mutation at position 125 also affected receptor binding, which was only observed when using recombinant H5 protein.

Subsequently, we analyzed the effect of the mutations in H5 on the HA-NA balance by analyzing virion self-elution from LAMP1-coated sensors (Fig 2C and 2F) similarly as described above (Fig 1). Virus with substitution S223N in H5 (H5_223N_N1) displayed much faster self-elution than parental H5N1_432E_ virus. This self-elution was even faster than H5N1 WT virus, carrying the same NA (Fig 2C). Similarly, viruses carrying mutations at position 156 (H5_156T_N1_432E_) or 125 (H5_125H/N_N1_432E_) eluted much faster than virus carrying a wild type H5 combined with the same NA (H5N1_432E_) (Fig 2F). The faster self-elution of H5_223N_N1 and H5_156T_N1_432E_ compared to their respective parental virus is consistent with the higher titers of these two viruses on MDCK cells. We conclude that the mutations in HA affect the HA-NA balance as they cause faster virion self-elution from a receptor-coated surface as a result of reduced HA-receptor binding.

### The importance of the 2SBS for virion self-elution depends on HA binding avidity

The A156T mutation in HA was obtained in 5 out of 6 replicates in H5N1 WT, although more passages were required as for H5N1_432E_ (Fig 1B, S1 Table and S2 Table). We therefore also compared the influence of this mutation on virion self-elution using H5N1 WT and H5_156T_N1 viruses. Again, the additional N-glycan in the head domain resulted in faster virion self-elution (Fig 2G). The selection of this mutation thus indicates that the HA-NA balance of H5N1 WT, which is presumably well adapted to replication in chickens, is not optimal for virus replication in MDCK cells, although this virus appears less off-balance than H5N1 with a mutated 2SBS (H5N1_432E_)(Fig 1A). The selection of H5_223N_N1 virus, which eluted even faster than H5N1 WT virus (Fig 2C), upon passaging of H5N1_432E_ is in agreement herewith. In some passage series, viruses were selected with mutations in HA without restoration of the 2SBS in NA. We therefore also analyzed the importance of the 2SBS on virion self-elution when NA was combined with a weaker binding H5_156T_ protein (Fig 2H). As expected, H5_156T_N1 virus eluted somewhat faster than H5_156T_N1_432E_, however, the importance of the 2SBS for self-elution was clearly smaller than when NA was combined with H5 lacking the additional glycan (compare Figs 1A and 2H). This prompted us to also analyze virus self-elution of H1N1 and H1N1_432E_ viruses, in which the same H5N1-derived N1 (with or without K432E substitution) is paired with HA of H1N1 strain PR8 (Fig 2I). Recombinant H1 protein displays reduced receptor binding when compared to H5 (S5 Fig). These H1N1 viruses were genetically stable [5] and passaging did not result in compensatory mutations. Virus with a functional 2SBS (H1N1) eluted somewhat faster than virus with a mutated 2SBS (H1N1_432E_)(Fig 2I), but again the difference between these two viruses was much smaller when compared to viruses carrying a much stronger-binding H5 (Fig 1A). We conclude that the contribution of the 2SBS to the HA-NA balance is smaller when viruses contain a weaker-binding HA.

### The effect of compensatory mutations in HA on receptor binding specificity

**Fig 3:**
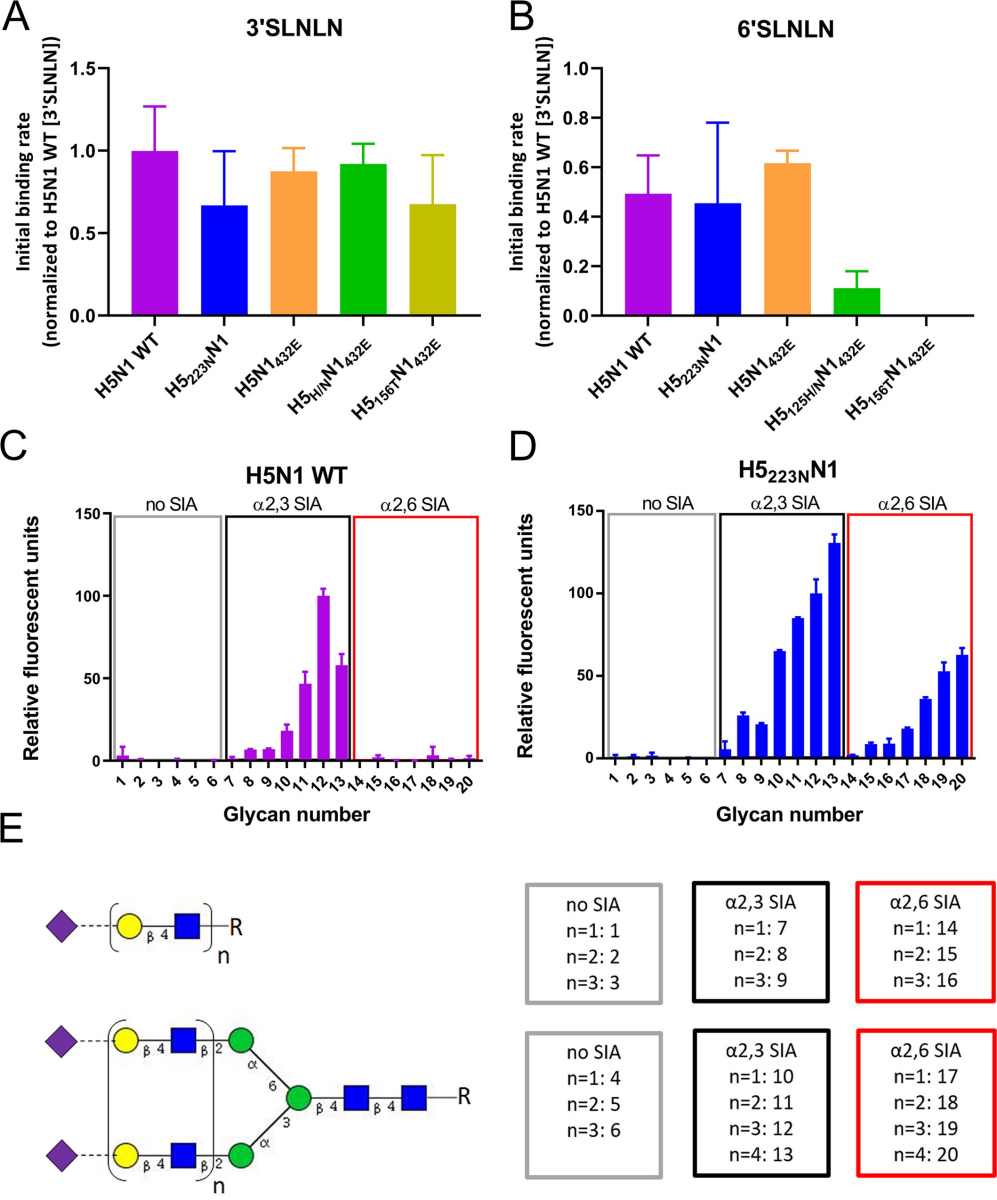
Mutation in HA affect virus receptor-binding specificity. (A and B) Initial binding rates of different H5N1 viruses for 3’SLNLN- (A) and 6’SLNLN- (B) coated sensors were determined as described previously [20, 24] and graphed normalized to the initial binding rate of H5N1 WT virus to 3’SLNLN. Means of three experiments are shown, standard deviations are indicated. (C and D) Glycan microarray analysis of the receptor binding specificities of H5N1 and H5_223N_N1 viruses. The relative fluorescent units normalized to glycan number 12 are graphed. The mean signals and standard deviations are shown for each glycan. The numbering of the glycans corresponds to the numbering of the glycans shown in (E). (E) Overview of the synthetic glycans printed on the microarray. Linear or branched glycans contain either no SIA, α2,3 SIA or α2,6 SIA and differ in their number of LAcNAc (N-acetyllactosamine [Galβ1-4GlcNAc]) repeats, indicated by n. Purple diamonds; SIA, yellow circles; Gal, blue squares; GlcNAc, green circles; Man.

The S223N mutation in H5 has been shown by some, but not by other studies to increase binding to human-type receptors [25–30]. We therefore studied to what extent the compensatory mutations in H5 affected receptor-binding specificity in the virus used in this study. To this end, we analyzed the initial binding rates of the viruses to BLI sensors (Fig 3A and 3B) coated with avian- (3’SLNLN; NeuAcα2-3Galβ1-4GlcNAcβ1-3Galβ1-4GlcNAc) or human-type (6’SLNLN; NeuAcα2-6Galβ1-4GlcNAcβ1-3Galβ1-4GlcNAc) receptors. The viruses displayed similar initial binding rates to 3’SLNLN (Fig 3A), but differed in their ability to bind 6’SLNLN (Fig 3B). Severely reduced binding to 6’SLNLN for H5_125H/N_N_1432E_ and H5_156T_N_1432E_ viruses was observed when compared to the control virus H5N1_432E_ with the same NA (Fig 3B). The relative receptor-binding specificity did not appear to be affected, however, by S223N (compare H5_223N_N1 and H5N1 WT; Fig 3A and 3B). Although the S223N mutation did not increase binding to 6’SLNLN in the BLI assay, this mutation may positively affect binding to other glycans containing α2,6-linked SIAs. Therefore, we probed the receptor specificity of H5N1 WT and H5_223N_N1 viruses using glycan array analysis. Both viruses preferred binding to branched glycans containing multiple LacNAc repeats capped with α2,3-linked SIAs (Fig 3C–3E). The H5_223N_N1, but not the H5N1 WT virus, displayed binding to the same glycans capped with α2,6-linked SIAs. Of note, antibody-complexed recombinant soluble H5 proteins, which are often used in glycan array analysis only bound to avian-type receptors (S6 Fig). We conclude that the S223N mutation results in increased binding to human-type receptors, which can only be observed for specific receptors and when using virus particles.

**Fig 4:**
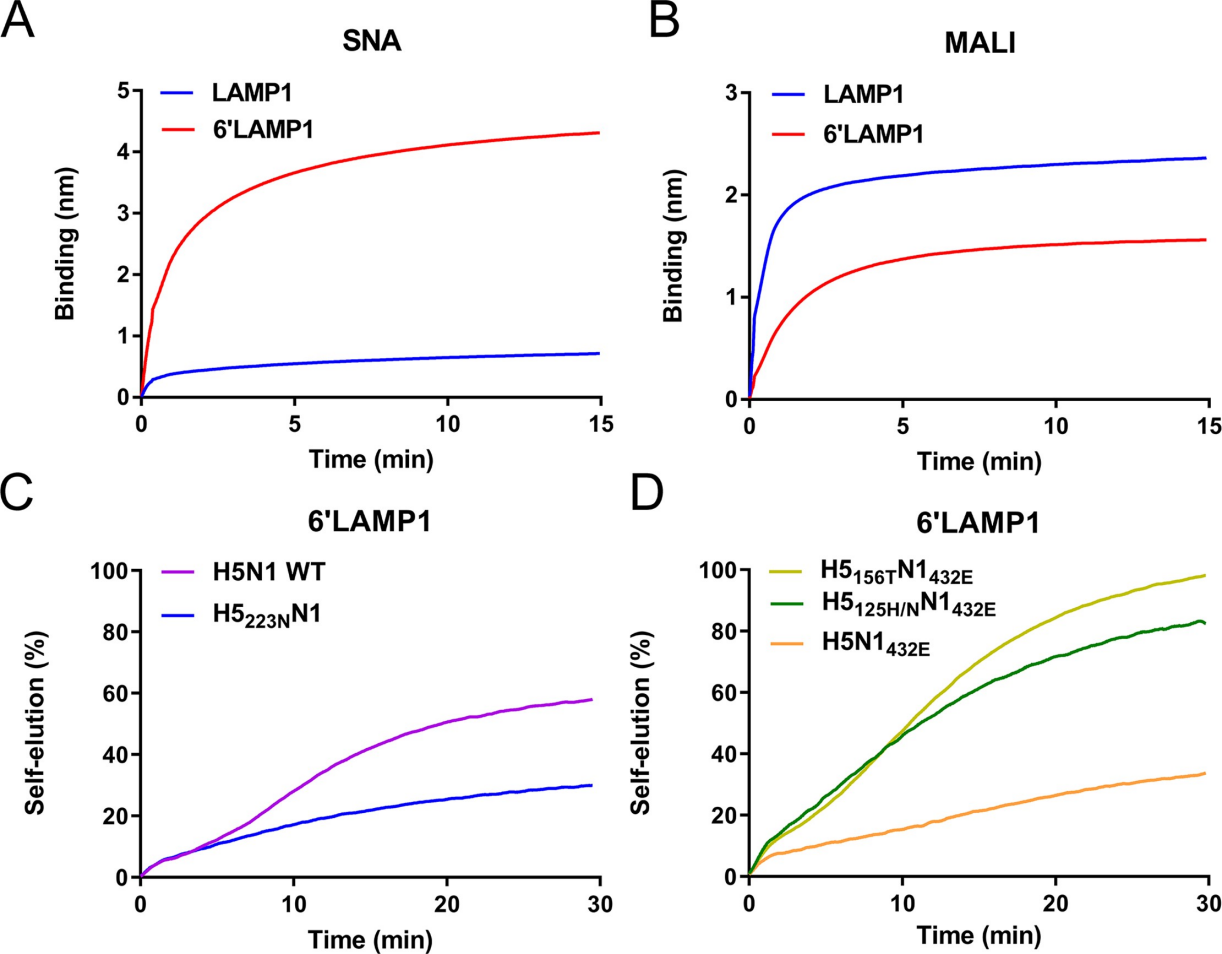
Mutation S223N in HA negatively affects self-elution from LAMP1 containing increased levels of α2,6 SIAs (6’LAMP1). (A and B) LAMP1 and 6’Lamp1 were analyzed for their attached glycans by BLI analysis of lectin binding to sensors coated with these glycoproteins as described previously [20]. (A) Binding of SNA, which is specific for SIAα2,6Galβ1,4GlcNAc oligosaccharide abundantly present on N-linked glycans. (B) Binding of MAL I, which is specific for SIAα2,3Galα1,4GlcNAc oligosaccharide abundantly present on N-linked glycans. We conclude that 6’LAMP1 contains increased levels of α2,6 SIAs and decreased levels of α2,3 SIAs. (C and D) NA-driven self-elution from 6’LAMP-coated sensors was analyzed for the indicated viruses (same stocks and amounts as used in in Fig 2) as described in the legend to Fig 1. Representative experiments out of 2–3 performed are shown. Mutation S223N results in faster self-elution from LAMP1 (Fig 2C), while elution from 6’LAMP is decreased.

Based on these results, we predicted that virus containing the S223N mutation should self-elute more slowly than its wild type counterpart from glycoprotein receptors containing high amounts of α2,6-linked SIAs. To test this hypothesis, we made use of recombinant LAMP1 containing increased levels of α2,6 SIA (6’LAMP1) by co-expression of human beta-galactoside alpha-2,6-sialyltransferase 1 (ST6Gal1). The presence of increased and decreased amounts of α2,6 and α2,3 SIAs on 6’LAMP1 compared to ‘standard’ LAMP1 was confirmed by binding of lectins specific for α2,6 (SNA) or α2,3 (MALI) SIAs (Fig 4A and 4B). As predicted, H5_223N_N1 eluted much slower from 6’LAMP1-coated sensor compared to H5N1 WT (Fig 4C), in contrast to results obtained for these viruses with LAMP1 (Fig 2C). As a control, we analyzed the self-elution of H5_156T_N1_432E_ and H5_125H/N_N1_432E_ viruses from 6’LAMP1. In agreement with the reduced binding to 6’SLNLN (Fig 3A), these viruses exhibited faster self-elution compared to H5N1_432E_ (Fig 4D).

### Selection of compensatory mutations in field viruses upon mutation of the 2SBS

**Fig 5:**
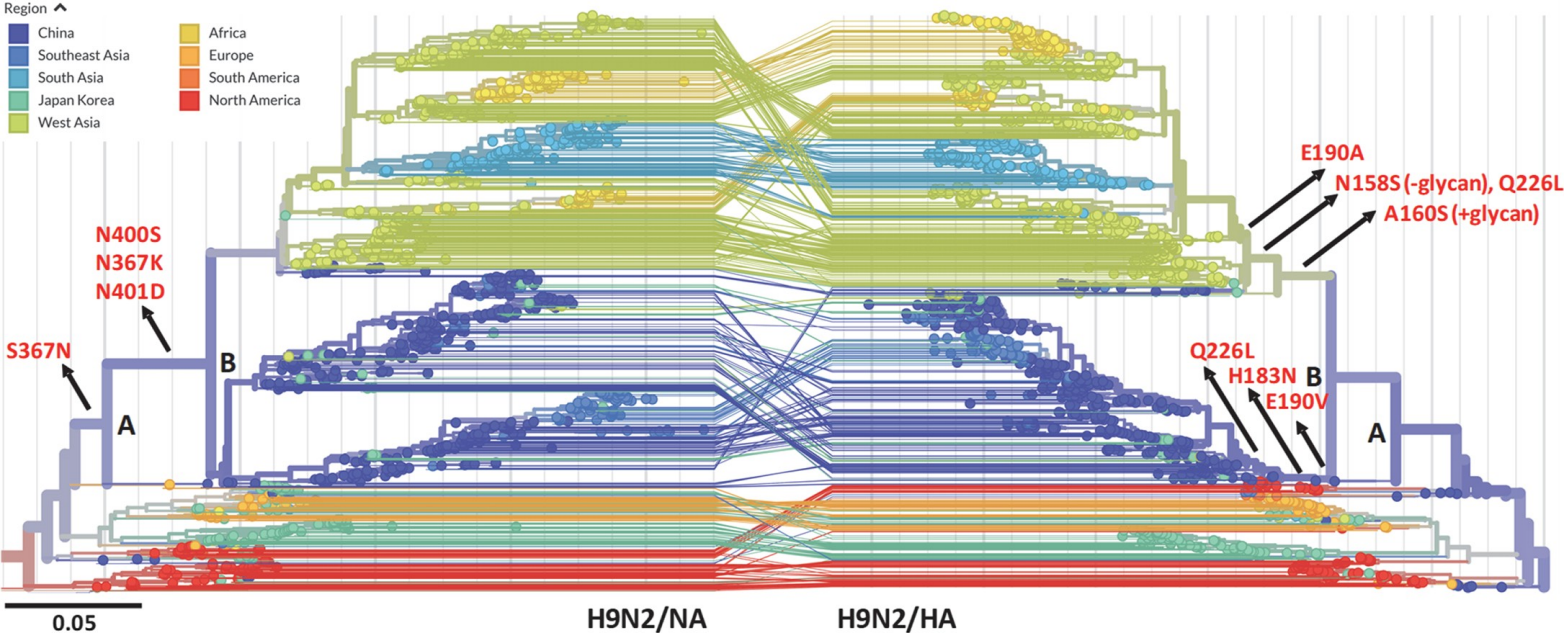
Mutation of the 2SBS in N2 precedes mutation of the RBS in H9 of H9N2 field strains. Phylogenetic trees of full-length NA and HA sequences of H9N2 viruses between 1970 and 2019 were generated using Nextstrain (https://nextstrain.org) [32]. Viruses are colored according to geographic location. NA (left) and HA (right) trees have a similar overall topology. Two corresponding branch points in the NA and HA trees are indicated by letters (A and B). Mutations in the 2SBS of NA or in HA that affect receptor binding are indicated, as well as the parts of the trunk of the trees to which they belong. Lines connecting HA and NA genes of the same isolate are shown. N2 and H3 numbering is used. Residue numbers shown for HA, correspond to residue number +8 in Nextstrain.

Our results indicate that mutation of the 2SBS may drive the selection of mutations in HA that reduce the avidity of HA-receptor binding and may affect HA receptor-binding specificity. The 2SBS in NA is highly conserved in avian IAVs with notable exceptions in H9N2 viruses [3]. Current H9N2 viruses, which are the most abundant IAVs found in poultry, also contain a mutated 2SBS accompanied by several mutations in HA that were reported to increase binding to human-type receptors (e.g. E190A/V, Q226L) [3, 31]. To determine the order, in which these mutations in NA and HA appeared in H9N2 viruses, phylogenetic analysis, using Nextstrain analytic and visualization tools (https://nextstrain.org)[32](Fig 5), was employed. The similar topology of the HA and NA gene trees marks the coevolution of these genes in the distinct clades indicated by different colors in Fig 5. Key mutations affecting the 2SBS of NA and the RBS of HA, occurring along the trunk of the trees, are indicated to show that mutations in the 2SBS occur just prior to the branchpoint (B) at the origin of the Chinese/East Asian and the West/South Asian and African clades. On the contrary, mutations in or near the RBS of HA occur after branchpoint B independently in both clades. Mutations in the first and second loop of the 2SBS include amino acid substitutions at positions known to interact with SIA (S367N/K and N400S) [17, 18]. Substitutions in the RBS of HA occur at positions known to affect receptor specificity (E190A/V and Q226L)[3, 31], accompanied in the West Asian clade by the gain (A160S) and subsequent loss (N158S) of a glycosylation site at the tip of the HA head domain. Of note, the Chinese/East Asian clade also acquired a mutation at the extremely conserved base of the RBS (H183N), which is expected to affect the receptor-binding properties of HA. In conclusion, mutations in the 2SBS of H9N2 NA preceded selection of mutations in HA that decrease and increase binding to avian- and human-type receptors, respectively.

## Discussion

We here show that mutation of the 2SBS in H5N1 NA, resulting in reduced NA activity, disturbs the HA-NA balance of virions, particularly when NA is accompanied by a strong-binding HA. Passaging such viruses rapidly resulted in the selection of mutations in NA and/or HA that restore the HA-NA balance providing further evidence for the importance of a precisely tuned balance for optimal replication and for an important role of the 2SBS therein. Mutations in HA, which resulted in higher virus titers particularly at early time points, reduced receptor-binding avidity either because they affect the RBS directly (S223N and H125N) [25–28, 33], or by resulting in an additional glycan in the head domain that possibly sterically hinders access to the RBS [34–37]. The additional glycan was eventually also observed upon passaging of H5N1 WT virus. Apparently, also the wild type virus used in this study did not display an HA-NA balance optimal for replication in MDCK cells, although its particular balance presumably allows efficient replication in chickens. When the 2SBS-positive/negative N1 proteins were combined with H1 of PR8 virus, genetically stable viruses were obtained [5], which is explained by the absence or presence of a functional 2SBS being much less critical for the HA-NA balance when NA is combined with a weak-binding HA. This also explains why H5N1 viruses with a mutated 2SBS did not repair their 2SBS after obtaining H125N or the additional glycan in the head domain. In agreement herewith, for H3N2 viruses we previously showed that the importance of the 2SBS for the HA-NA balance and virus replication depends on the receptor-binding properties of HA with which NA is combined. A human H3-containing virus replicated faster when it contained a 2SBS-negative N2 rather than a 2SBS-positive N2. The opposite was observed for an avian H3-containing virus [20].

As H5 prefers binding to avian-type receptors, mutations in HAs, upon disruption of the 2SBS, are primarily selected on their ability to reduce binding to these receptors. Mutation S223N, however, simultaneously resulted in increased binding to human-type receptors. This mutation has previously been observed in H5N1 viruses isolated from humans [25, 38] and has been shown by some, but not by other studies to increase binding to human-type receptors [25–30]. Differences in human-type receptor-binding abilities for N223-containing HAs may be explained by different genetic backgrounds (e.g. absence or presence of a glycan at position 160) and/or experimental assays used. The H5N1 virus that acquired N223 in this study displays increased binding to several, but not all, human-type receptors using glycan array analysis, while this was not observed when recombinant H5 protein was used. Apparently, low multivalency of antibody-HA complexes compared to virus particles prevented detection of low-affinity binding to human-type receptors. Thus, low affinity receptors may be missed when performing glycan array analyses with recombinant proteins rather than viruses [12]. Our glycan array data are corroborated by the reduced self-elution of virus with the S223N mutation from a LAMP1 glycoprotein containing increased levels of α2,6 SIAs, as stronger HA binding will reduce self-elution. The other mutations that were selected upon passaging (H125N and A156T) did not result in enhanced binding to human-type receptors, but decreased binding to those even more so than to avian-type receptors. Thus, upon mutation of the 2SBS, mutations in HA are selected because they decrease binding to α2,3 SIAs. Concomitantly, these mutations can affect binding to human-type receptors either positively or negatively.

It is well established that all human viruses contain substitutions in the RBS of HA (e.g. Q226L) that increase binding to α2,6- linked SIAs and reduce binding to α2,3-linked SIAs, when compared to avian viruses [1, 2, 39–41]. In addition, the 2SBS, which is highly conserved in NA of avian viruses, is invariably lost in all human (pandemic) viruses [5, 16, 18, 20, 23]. Apparently, this particular combination of HA and NA features results in a HA-NA balance that allows optimal replication and spread in humans. Different scenarios might be envisioned by which avian viruses adapt their HA-NA balance to become pandemic viruses. On the one hand, mutations in HA, obtained e.g. when replicating in the new human host, may precede those in NA. The altered receptor-binding properties of HA may subsequently select for mutations in the 2SBS to obtain an optimal balance for replication in humans. Alternatively, avian viruses may also first acquire mutations in the 2SBS of NA, which may subsequently drive mutations in the RBS of HA that decrease receptor binding to avian-type receptors and accidentally increase binding to human-type receptors. The resulting viruses may more easily jump to humans, where the HA-NA balance may be adjusted further by additional mutations. Too few sequences are available of clinical isolates to conclude which scenario occurred in 1918 or 1957. In this study, we provide experimental evidence that disturbance of the 2SBS of NA may indeed select for mutations in HA that alter its receptor-binding properties, resulting in increased binding to human-type receptors. The plausibility of this scenario is supported by phylogenetic analyses that indicate that also for avian H9N2 viruses mutations in the 2SBS preceded, and may have driven, selection of mutations in the RBS of HA that reduce binding to avian-type receptors and increase binding to human-type receptors, and that may increase the zoonotic potential of these viruses. A similar scenario also appears to apply for novel H7N9 viruses, as a mutation in the 2SBS, albeit not a SIA-contact residue, that affected receptor binding and catalytic activity preceded a mutation in the RBS in HA (Q226L) [4], which reduced binding to avian-type receptors and increased binding to human-type receptors[2, 39, 42]. What drives mutation of the generally highly conserved 2SBS in NA of these avian viruses remains elusive, but might be related to adaptation of these viruses to a another avian host. Regardless, these observations warrant more detailed attention to the evolution of NA in avian viruses in relation to altered receptor-binding properties of these viruses.

## Materials and methods

### Recombinant viruses

Recombinant viruses were generated in the background of A/Puerto Rico/8/34 H1N1 (PR8 H1N1) by means of reverse genetics, using PR8 plasmids provided by drs Hoffmann and Webster (St. Jude Children's Research Hospital, Memphis) as described [43, 44]. Recombinant H1N1 and H5N1 viruses contain the NA gene (GenBank accession no. BAM85820.1) from A/duck/Hunan/795/2002(H5N1) combined with either the HA gene of PR8 (7+1 virus) or its cognate HA gene (Accession number CY028963; 6+2 virus) [5]. The 2SBS in NA was mutated by replacing the AAA codon encoding K432 in wild type N1 by the GAG codon encoding E432 in N1_432E_. The nucleotide sequence encoding the wild-type multi-basic amino-acid sequence in H5 was modified to encode a low-pathogenic cleavage site as described previously [44]. The recombinant viruses were rescued in MDCK-II (ATCC) cells.

### Passaging of recombinant viruses and sequence analysis of HA and NA genes

After rescue of the viruses in MDCK-II cells, the virus stocks were immediately used in the passage experiments. Viruses were passaged on MDCK-II cells at a multiplicity of infection (MOI) of 0.001 tissue culture infectious dose 50 (TCID50) per cell. After about 44hpi, cells culture media of infected cells were harvested and titers were determined by endpoint titration in MDCK-II cells. For each passage, viral RNA was extracted from cell culture media of infected cells with NucleoSpin RNA virus kit (Macherey-Nagel) according to the manufacturer’s instructions and converted to cDNA using SuperScript III Reverse Transcriptase (invitrogen) with random Hexamers (invitrogen). HA and NA sequences were amplified using the Q5 High-Fidelity DNA Polymerase (NEB) using specific primers (HA: forward: 5’- ATGGAGAAAATAGTGCTTCTTCTTG CAA-3’, reverse: 5’-TTCTGCATTGTAACGATCCATTGGA-3’; NA: forward: 5’-TGAATCCAAATCAGAAGATAATAACCATCG-3’, reverse: 5’- GTCAATGGTGAATGGCAACTCAGCA-3’). PCR products were sized-separated using agarose gel electrophoresis and purified by NucleoSpin Gel and PCR Clean-upkit (Macherey-Nagel) following manufacturer’s instructions and then analyzed by Sanger sequencing (Macrogen).

### Expression of recombinant proteins

Human-codon optimized cDNAs encoding the H1 ectodomain of A/Puerto Rico/8/34/Mount Sinai (H1N1)(GenBank accession no. AF389118.1) or H5 ectodomain of A/duck/Hunan/795/2002(H5N1) (GenBank accession no. CY028963.1) were cloned into pFRT or pCD5 expression plasmids as described previously [45]. Expression plasmids carrying the H5 S223N, H125N and A156T were made by using site directed mutagenesis. Human-codon optimized recombinant soluble N1 expression constructs were described previously [5]. Expression constructs encoding full length N1 were obtained by replacement of the signal sequence-, Strep tag- and Tetrabrachion tetramerization domain-encoding cDNAs in these constructs with cDNA encoding the N-terminal part of N1 (cytoplasmic tail, transmembrane domain and part of the stalk domain). Generation of pCAGGs vector containing codon-optimized glycoprotein LAMP1 ectodomain-encoding cDNAs genetically fused to sequences encoding a Fc-tag, for Protein-A based purification, and a Bap tag [46], for binding to octet sensors, was described previously [24]. Generation of a pCAGGs vector encoding antibody Fi6v3 [47], was performed similarly as described previously [48]. HA plasmids were transfected into HEK293S GnTI(-) [49, 50] cells, while NA, Fi6 and LAMP1 expression plasmids were transfected into HEK293T (ATCC) cells, using polyethylenimine (PolyScience) [51]. An expression vector encoding BirA ligase was cotransfected with the LAMP1-coding vectors, and an expression vector encoding human ST6Gal1 was also included to get 6’LAMP1 [24]. Five days post transfection, cell culture media containing soluble HA, NA and glycoproteins were harvested and purified using Streptactin- or protein A- containing beads [24, 51]. Purified HA and NA were quantified by quantitative densitometry of GelCode Blue (Thermo Fisher Scientific)-stained protein gels additionally containing bovine serum albumin (BSA) standards. The signals were imaged and analyzed with an Odyssey imaging system (LI-COR). N1-containing VLPs released into the cell culture media upon expression of full length N1 were harvested after 3 days post-transfection. The amount of N1 in these preparations was determined by performing a MUNANA assay using limiting dilutions of the VLP preparations similarly as described previously for N2 [20].

### Biolayer interferometry (BLI) assays

BLI assays were performed using the Octet RED348. All the experiments were performed in Dulbecco's phosphate buffered saline (PBS) with Calcium and Magnesium (Lonza) at 30°C and with plates shaking at 1000 rpm. Streptavidin sensors were loaded to saturation unless indicated otherwise with biotinylated synthetic glycans 3’SLNLN, 6’SLNLN, LNLN, or with LAMP1 or 6’LAMP1 glycoproteins. Synthetic glycans were synthesized at the Department of Chemical Biology and Drug Discovery, Utrecht University, Utrecht, the Netherlands and the Complex Carbohydrate Research Center, University of Georgia, Athens, USA [52]. The virus binding and self-elution assays were performed as described previously [20, 24]. Virus particle numbers were determined by Nanoparticle tracking analysis as described previously [20]. Virus binding was monitored by moving receptor-loaded sensors to wells containing virus and OC (kindly provided by Roche). Relative initial binding rates (v_obs_ = dB/dT) per virus particle, which quantifies virus binding affinity, were calculated similarly as previously described [20, 24]. For analysis of NA-driven dissociation, viruses were loaded to same level in the presence of OC prior to the dissociation in the absence of OC. OC was removed by three short (5s) washes of the sensor in PBS. BLI assays with recombinant H5 proteins were carried out as described previously [53]. In short, similar amount of HA proteins complexed with strepMabClassic-HRP (IBA; 2:1 molar ratio) were applied in the HA binding assay. Binding was analyzed for 20 mins, followed by a dissociation phase in PBS. The kinetic NA activity assay was performed as described previously [20, 24], In brief, sensors loaded with synthetic glycans were incubated in buffer containing N1 in the absence or presence of Erythrina cristagalli lectin (ECA; Vector Labs) or ECA alone. ECA binding to sensors coated with 3’SLNLN or 6’SLNLN is a measure for SIA cleavage from these receptors by NA. Differential sialylation of LAMP1 and 6’LAMP was analyzed by incubation of glycoprotein-coated sensors with 80 μg/mL Sambuccus nigra elderberry bark lectin (SNA; Vector Labs) or with 80 μg/mL Maackia Amurensis Lectin I (MAL I; Vector Labs).

### Glycan array analysis

The 100 μM synthetic compounds in sodium phosphate buffer (250 mM, pH 8.5) were printed on activated glass slides (Nexterion Slide H, Schott Inc) by piezoelectric non-contact printing (sciFLEXARRAYER S3, Scienion Inc) with a drop volume of ~400 pL and 1 drop per spot at 50% relative humidity [54]. Compounds were printed as replicates of 6, with 32x25 spots per subarray and 24 subarrays (3x8) per slide. After overnight incubation in a saturated NaCl chamber (75% relative humidity), the remaining activated esters were quenched with ethanolamine (50 mM) in TRIS (100 mM, pH 9.0). Next, slides were rinsed with DI water, dried by centrifugation, and stored in a desiccator at RT. To validate printing, sub-arrays were incubated at RT with 50 μL mixtures of 10 μg/mL biotinylated lectins from Vector Labs (ECA, MAL II, and SNA) and 5 μg/mL Streptavidin-AlexaFluor635 (ThermoFisher Scientific) in TSM binding buffer (20 mM Tris Cl, pH 7.4, 150 mM NaCl, 2 mM CaCl2, 2 mM MgCl2, 0.05% Tween, 1% BSA) for 1 h followed by 4 successive washes of the whole slide with 1 time TSM wash buffer (20 mM Tris Cl, pH 7.4, 150 mM NaCl, 2 mM CaCl2, 2 mM MgCl2, 0.05% Tween-20), 1 time TSM buffer (20 mM Tris Cl, pH 7.4, 150 mM NaCl, 2 mM CaCl2, 2 mM MgCl2), 2 times DI water with each 10 min soak time. Recombinant HAs (H5, H5_156T_, H5_125N_, H5_223N_) were precomplexed at 50 μg/mL with StrepMAb-Classic (12.5 μg/mL; IBA) and goat anti-mouse IgG H&L-AlexaFluor647 (6.25 μg/mL; Abcam) in 50 μL TSM binding buffer. After incubation on ice for 30 min, the mixtures were added to the sub-arrays for 90 min. Washes were performed as described above for the plant lectins. Similarly as for the HAs, virus stocks (H5N1 and H5_223N_N1) were precomplexed with Fi6v3 antibody (5 μg/mL) and anti-human IgG-Cy3 (5 μg/mL; Jackson) in the presence of OC (10 μM). All wash steps were performed in the presence of OC (10 μM). Washed arrays were dried by centrifugation and immediately scanned for fluorescence on a GenePix 4000 B microarray scanner (Molecular Devices). The detection gain was adjusted to avoid saturation of the signal. The data were processed with GenePix Pro 7 software and further analyzed using our home written Microsoft Excel macro. After removal of the lowest and highest value of the 6 replicates, the mean fluorescent intensities (corrected for mean background) and standard deviations were calculated (n = 4). Data were fitted using Prism software (GraphPad Software, Inc).

## Supporting information

S1 Fig Detailed analysis of the importance of K432E mutation in NA on receptor binding and activity of recombinant proteins. (A) Hemagglutination assays were carried out using virus-like particle (VLP) preparations containing similar amounts of N1 protein similarly as described previously [20]. In short, VLPs were harvested from cells expressing full-length N1 proteins. Similar amounts of NA activity as determined using the monovalent substrate MUNANA were used in the analysis. Serial twofold dilutions of the VLPs were incubated in triplicate with equal volumes of 0.5% human erythrocytes at 4°C for 2 h in the presence or absence of OC. Red dots at the bottom of the wells indicate hemagglutination negative wells. N1 displayed a much higher hemagglutinating activity than N1432E. In the absence of OC, the difference in hemagglutination was even larger (S1A Fig), presumably because the receptor density on the red blood cells is decreased by NA activity and a much higher receptor density is need for hemagglutination with N1432E than with N1 (B) Analysis of NA enzymatic activity by BLI kinetic assay was performed similarly as described previously [20]. Briefly, streptavidin biosensors were coated with biotinylated synthetic glycans (3’SLNLN, 6’SLNLN or LNLN). Subsequently, the sensors were incubated in buffer containing 4 μg recombinant soluble N1 or N1432E in the absence or presence of 8 μg ECA or ECA alone. ECA binding to sensors coated with 3’SLNLN or 6’SLNLN is a measure for SIA cleavage from these receptors by NA. Experiments were independently performed three times with similar results. Representative experiments are shown. N1 displays a higher enzymatic activity then N1432E, which is only observed for 3’SLNLN. No appreciable cleavage of 6’SLNLN is observed.
(TIF)

S2 Fig Sequence analysis of HA and NA genes upon H5N1 virus passaging. Examples of sequencing results are shown. (A) Sequencing results obtained for NA of H5N1_432E_ passaging series A1 (S1 Table). (B) Sequencing results obtained for HA of H5N1_432E_ passaging series A1 and B2. Mutated residues and passage numbers are indicated.
(TIF)

S3 Fig Sequence of H5. Sequence of H5 protein of A/duck/Hunan/795/2002(H5N1) used in this study is shown. Residues that were mutated in this study are colored magenta (H125N, A156T and S223N). Amino acids of the three structural elements (130-loop, 190-helix, 220-loop) of RBD are shown in yellow. The four conserved, structurally-important amino acids of RBS are labelled with asterisks. Mutations A156T and A156S result in a N-glycosylation consensus sequence (NXS/T, X is any amino acid except P).
(TIF)

S4 Fig Glycosylation of recombinant HAs. (A) Recombinant soluble H5 proteins expressed in HEK293S GnTI(-) were analyzed by gel electrophoresis followed by GelCode Blue staining. H5_156T_ runs at a higher position in the gel than the other H5 proteins. (B) After (mock) treatment of H5 with PNGase F, the recombinant soluble H5 and H5_156T_ proteins were examined by gel electrophoresis and GelCode Blue staining. The difference in electrophoretic mobility of the H5 proteins is lost upon removal of the N-glycans with PNGase F, indicating that H5_156T_ contains an additional N-glycan side chain compared to H5. The position in the gel of relevant molecular weight markers is shown on the right side of the gels.
(TIF)

S5 Fig Receptor-binding avidity of H1 and H5 proteins. Binding of HA to fetuin was analyzed using a fetuin solid phase binding assay as described previously [45, 53]. Briefly, purified, soluble trimeric HAs were precomplexed with strepMabClassic-HRP and rabbit-α-mouse-HRP (4:2:1 molar ratio) prior to incubation of limiting dilutions on the fetuin-coated (100μg/ml fetuin per well) 96-well Nunc MaxiSorp plates. After one hour incubation at room temperature, HA binding was subsequently determined using tetramethylbenzidine substrate (TMB, bioFX) in ELISA reader EL-808 (BioTEK) by measuring the optical density at 450 nm (OD450), which corresponds to binding of HA to fetuin. Standard deviations (n = 3) are indicated. H5 displayed a much higher receptor-binding avidity than H1. Low level expression of H1 precluded a BLI-based assay as shown in Fig 2A, for which high levels of HA are needed.
(TIF)

S6 Fig Glycan microarray with recombinant soluble H5 proteins. Glycan microarray analysis was used to determine the receptor binding specificities of recombinant soluble H5 (A), H5156T (B), H5125N (C) and H5223N (D). All the HAs were precomplexed with antibodies against the Strep tag and goat anti-mouse IgG H&L (Alexa Fluor 647) similarly as described previously [52, 55]. The mean signals and standard deviations are shown for each glycan. The numbering of the glycans corresponds to the numbering of the glycans shown in (E). (E) Overview of the synthetic glycans printed on the microarray. Linear or branched glycans contain either no SIA, α2,3 SIA or α2,6 SIA and differ in their number of LAcNAc (N-acetyllactosamine [Galβ1-4GlcNAc]) repeats, indicated by n. Purple diamonds; SIA, yellow circles; Gal, blue squares; GlcNAc, green circles; Man.
(TIF)

S1 Table Sequence analysis of 2SBS-negative H5N1_432E_ virus upon passaging. (TIF)

S2 Table Sequence analysis of 2SBS-positive H5N1_432K_ virus upon passaging. (TIF)

S3 Table Overview of virus stocks used. (TIF)
