# Supplementary figures and images for: Mutation of the second sialic acid-binding site of influenza A virus neuraminidase drives compensatory mutations in hemagglutinin
Source: PLoS Pathog. 2020 Aug 27;16(8):e1008816. doi: 10.1371/journal.ppat.1008816 (PMC7480853; doi:10.1371/journal.ppat.1008816)

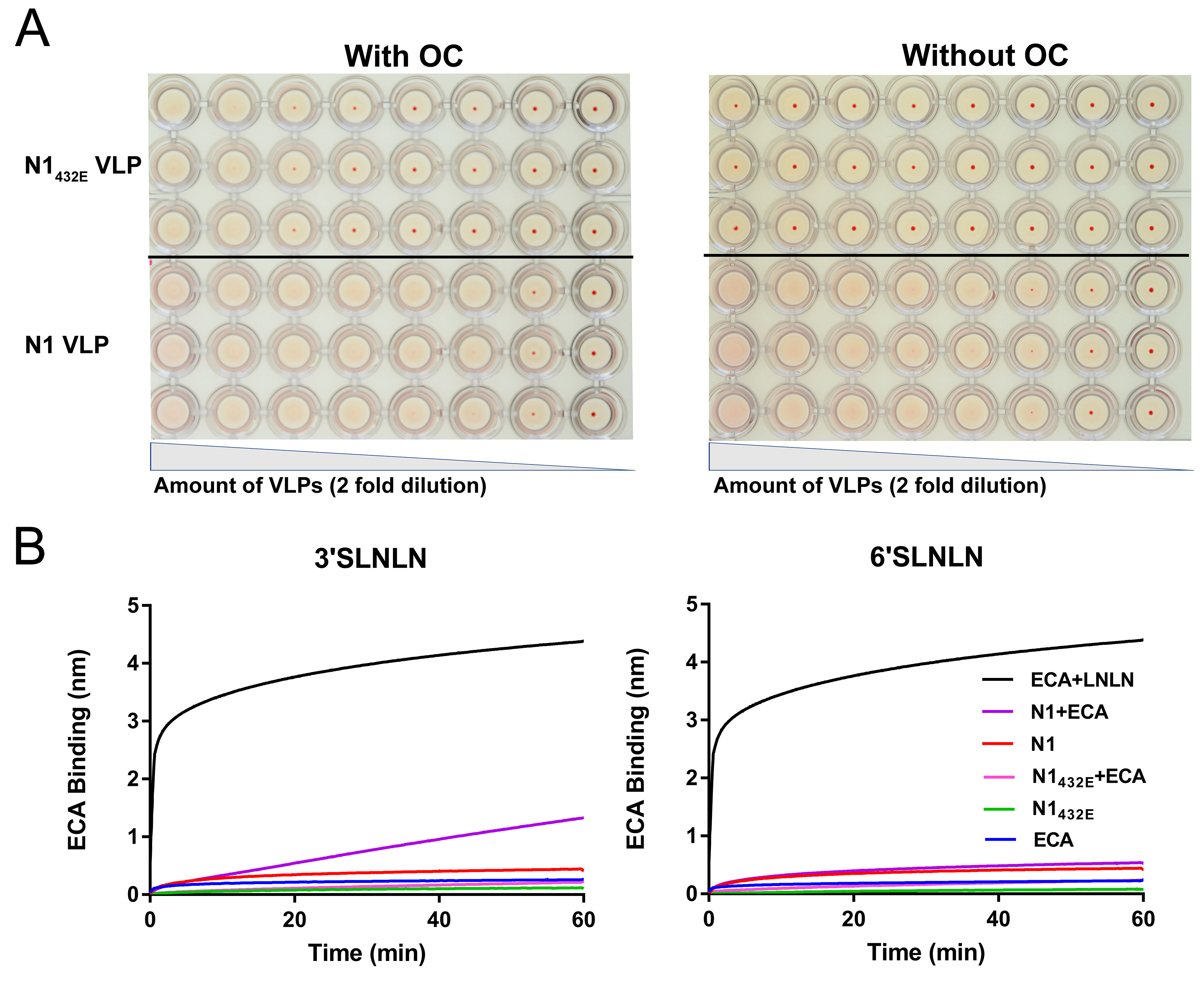

Supplement: S1 Fig — (A) Hemagglutination assays were carried out using virus-like particle (VLP) preparations containing similar amounts of N1 protein similarly as described previously [20]. In short, VLPs were harvested from cells expressing full-length N1 proteins. Similar amounts of NA activity as determined using the monovalent substrate MUNANA were used in the analysis. Serial twofold dilutions of the VLPs were incubated in triplicate with equal volumes of 0.5% human erythrocytes at 4°C for 2 h in the presence or absence of OC. Red dots at the bottom of the wells indicate hemagglutination negative wells. N1 displayed a much higher hemagglutinating activity than N1432E. In the absence of OC, the difference in hemagglutination was even larger (S1A Fig), presumably because the receptor density on the red blood cells is decreased by NA activity and a much higher receptor density is need for hemagglutination with N1432E than with N1 (B) Analysis of NA enzymatic activity by BLI kinetic assay was performed similarly as described previously [20]. Briefly, streptavidin biosensors were coated with biotinylated synthetic glycans (3’SLNLN, 6’SLNLN or LNLN). Subsequently, the sensors were incubated in buffer containing 4 μg recombinant soluble N1 or N1432E in the absence or presence of 8 μg ECA or ECA alone. ECA binding to sensors coated with 3’SLNLN or 6’SLNLN is a measure for SIA cleavage from these receptors by NA. Experiments were independently performed three times with similar results. Representative experiments are shown. N1 displays a higher enzymatic activity then N1432E, which is only observed for 3’SLNLN. No appreciable cleavage of 6’SLNLN is observed. (TIF) [file ppat.1008816.s001.tif]

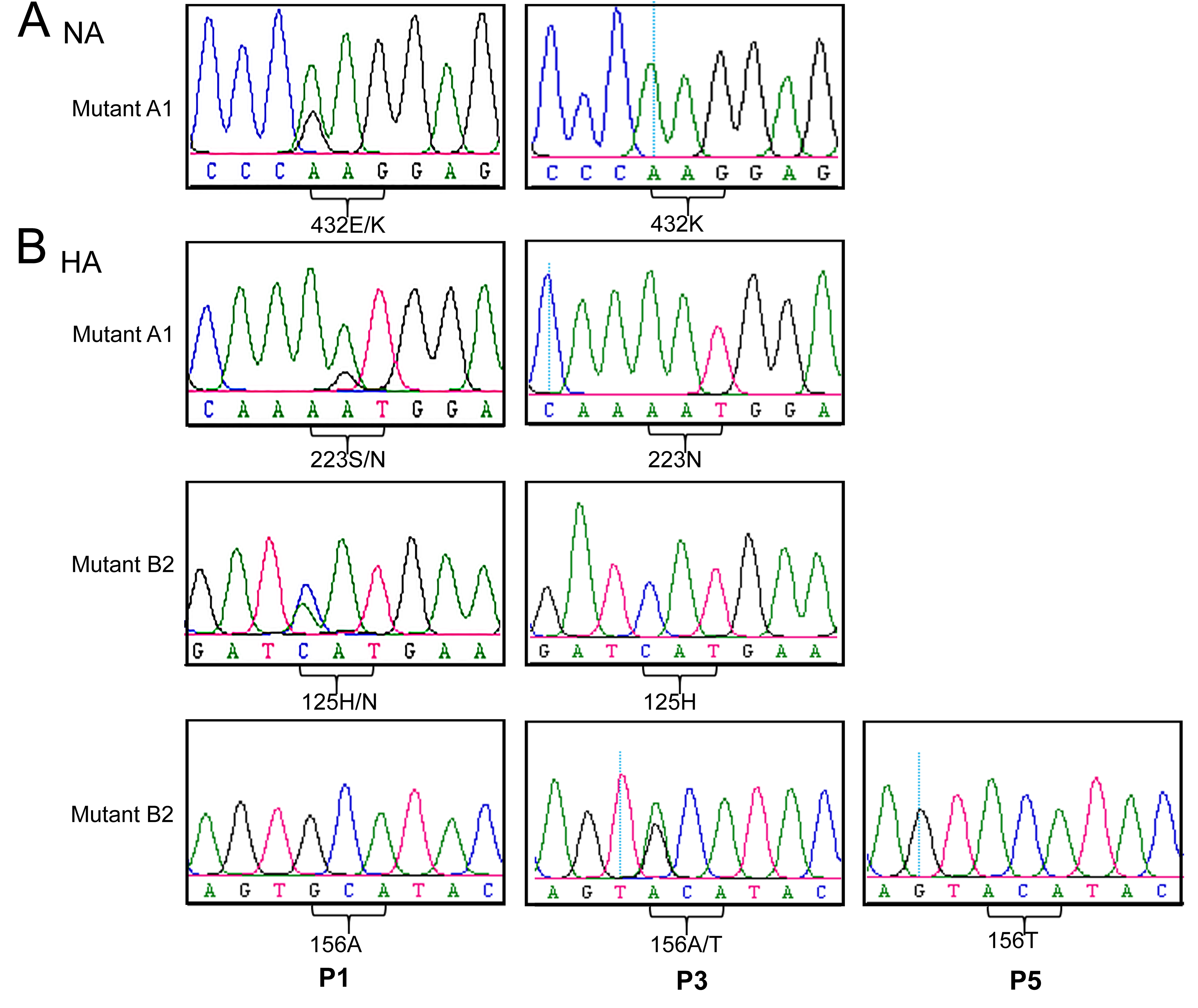

Supplement: S2 Fig — Examples of sequencing results are shown. (A) Sequencing results obtained for NA of H5N1432E passaging series A1 (S1 Table). (B) Sequencing results obtained for HA of H5N1432E passaging series A1 and B2. Mutated residues and passage numbers are indicated. (TIF) [file ppat.1008816.s002.tif]

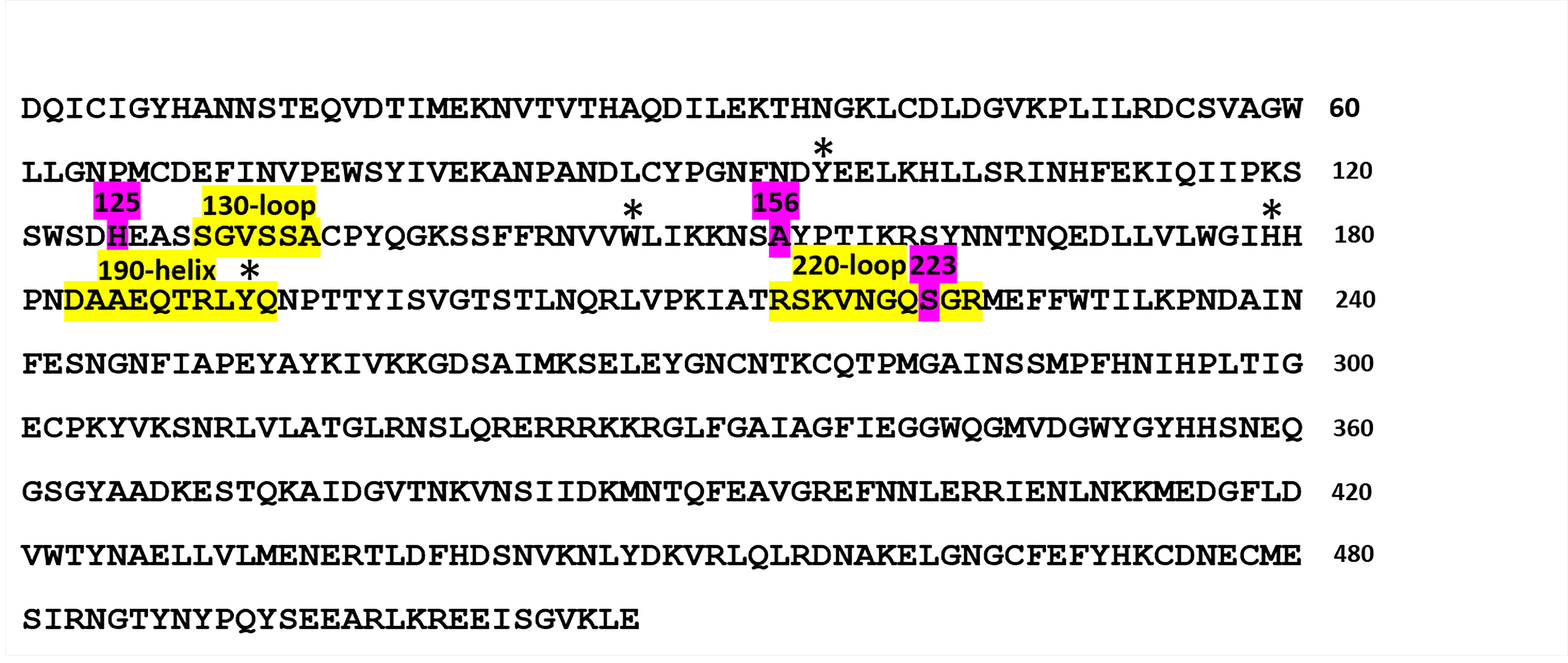

Supplement: S3 Fig — Sequence of H5 protein of A/duck/Hunan/795/2002(H5N1) used in this study is shown. Residues that were mutated in this study are colored magenta (H125N, A156T and S223N). Amino acids of the three structural elements (130-loop, 190-helix, 220-loop) of RBD are shown in yellow. The four conserved, structurally-important amino acids of RBS are labelled with asterisks. Mutations A156T and A156S result in a N-glycosylation consensus sequence (NXS/T, X is any amino acid except P). (TIF) [file ppat.1008816.s003.tif]

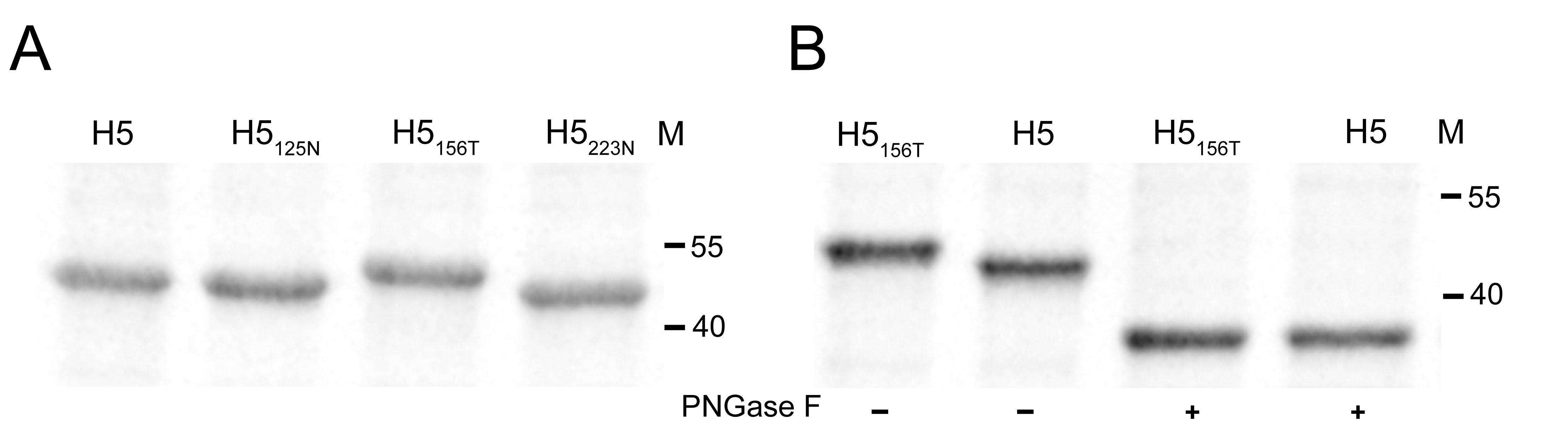

Supplement: S4 Fig — (A) Recombinant soluble H5 proteins expressed in HEK293S GnTI(-) were analyzed by gel electrophoresis followed by GelCode Blue staining. H5156T runs at a higher position in the gel than the other H5 proteins. (B) After (mock) treatment of H5 with PNGase F, the recombinant soluble H5 and H5156T proteins were examined by gel electrophoresis and GelCode Blue staining. The difference in electrophoretic mobility of the H5 proteins is lost upon removal of the N-glycans with PNGase F, indicating that H5156T contains an additional N-glycan side chain compared to H5. The position in the gel of relevant molecular weight markers is shown on the right side of the gels. (TIF) [file ppat.1008816.s004.tif]

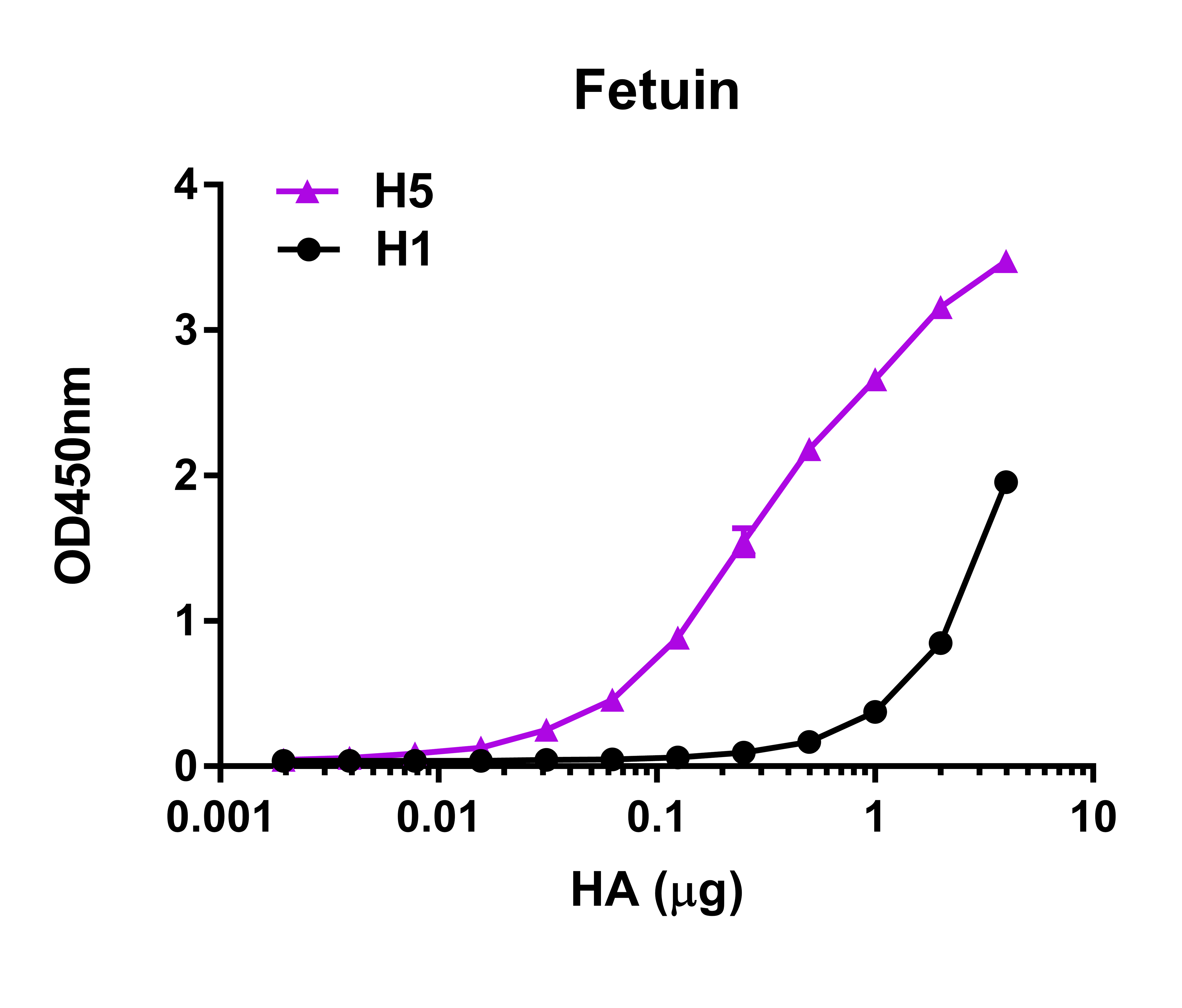

Supplement: S5 Fig — Binding of HA to fetuin was analyzed using a fetuin solid phase binding assay as described previously [45, 53]. Briefly, purified, soluble trimeric HAs were precomplexed with strepMabClassic-HRP and rabbit-α-mouse-HRP (4:2:1 molar ratio) prior to incubation of limiting dilutions on the fetuin-coated (100μg/ml fetuin per well) 96-well Nunc MaxiSorp plates. After one hour incubation at room temperature, HA binding was subsequently determined using tetramethylbenzidine substrate (TMB, bioFX) in ELISA reader EL-808 (BioTEK) by measuring the optical density at 450 nm (OD450), which corresponds to binding of HA to fetuin. Standard deviations (n = 3) are indicated. H5 displayed a much higher receptor-binding avidity than H1. Low level expression of H1 precluded a BLI-based assay as shown in Fig 2A, for which high levels of HA are needed. (TIF) [file ppat.1008816.s005.tif]

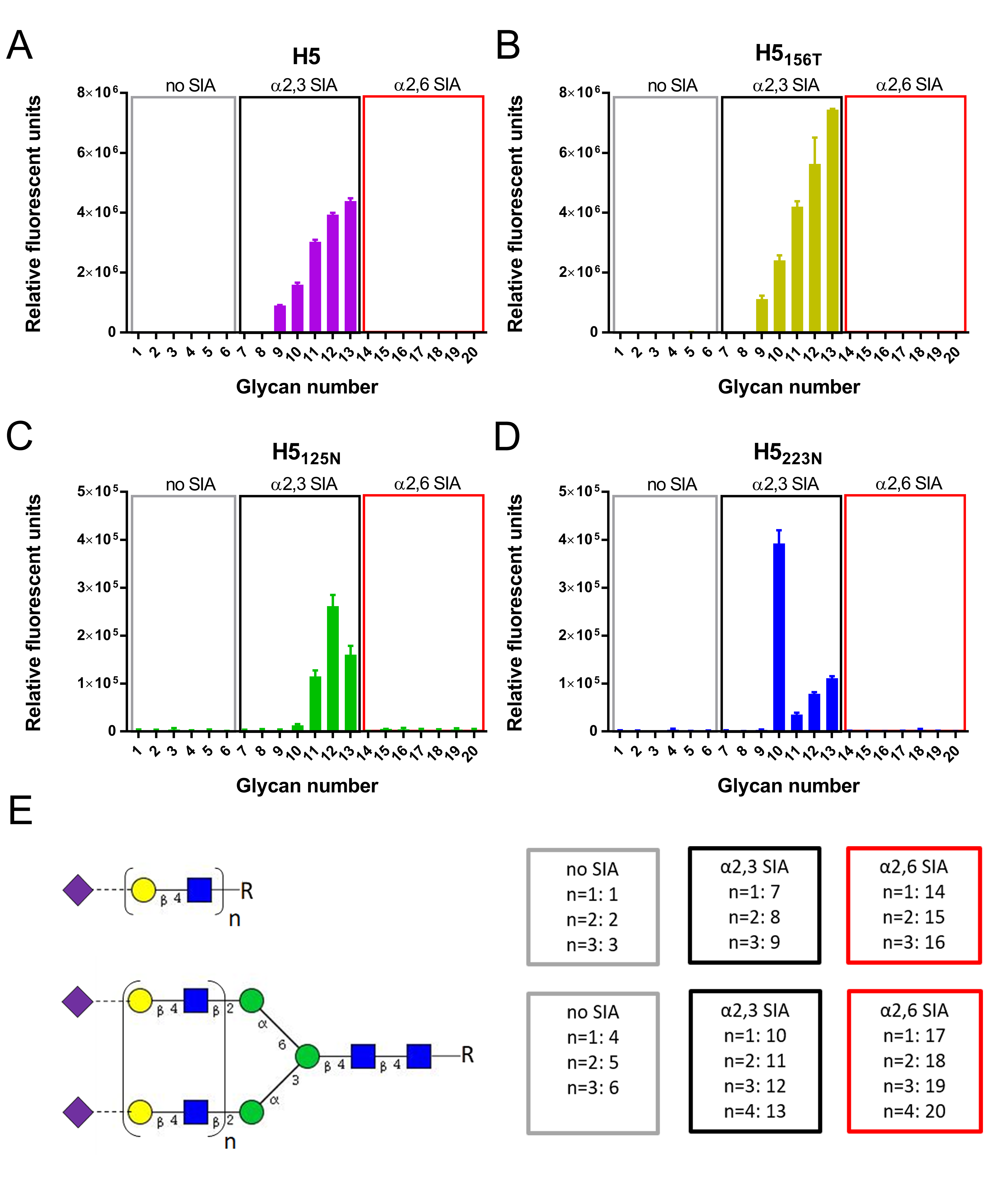

Supplement: S6 Fig — Glycan microarray analysis was used to determine the receptor binding specificities of recombinant soluble H5 (A), H5156T (B), H5125N (C) and H5223N (D). All the HAs were precomplexed with antibodies against the Strep tag and goat anti-mouse IgG H&L (Alexa Fluor 647) similarly as described previously [52, 55]. The mean signals and standard deviations are shown for each glycan. The numbering of the glycans corresponds to the numbering of the glycans shown in (E). (E) Overview of the synthetic glycans printed on the microarray. Linear or branched glycans contain either no SIA, α2,3 SIA or α2,6 SIA and differ in their number of LAcNAc (N-acetyllactosamine [Galβ1-4GlcNAc]) repeats, indicated by n. Purple diamonds; SIA, yellow circles; Gal, blue squares; GlcNAc, green circles; Man. (TIF) [file ppat.1008816.s006.tif]

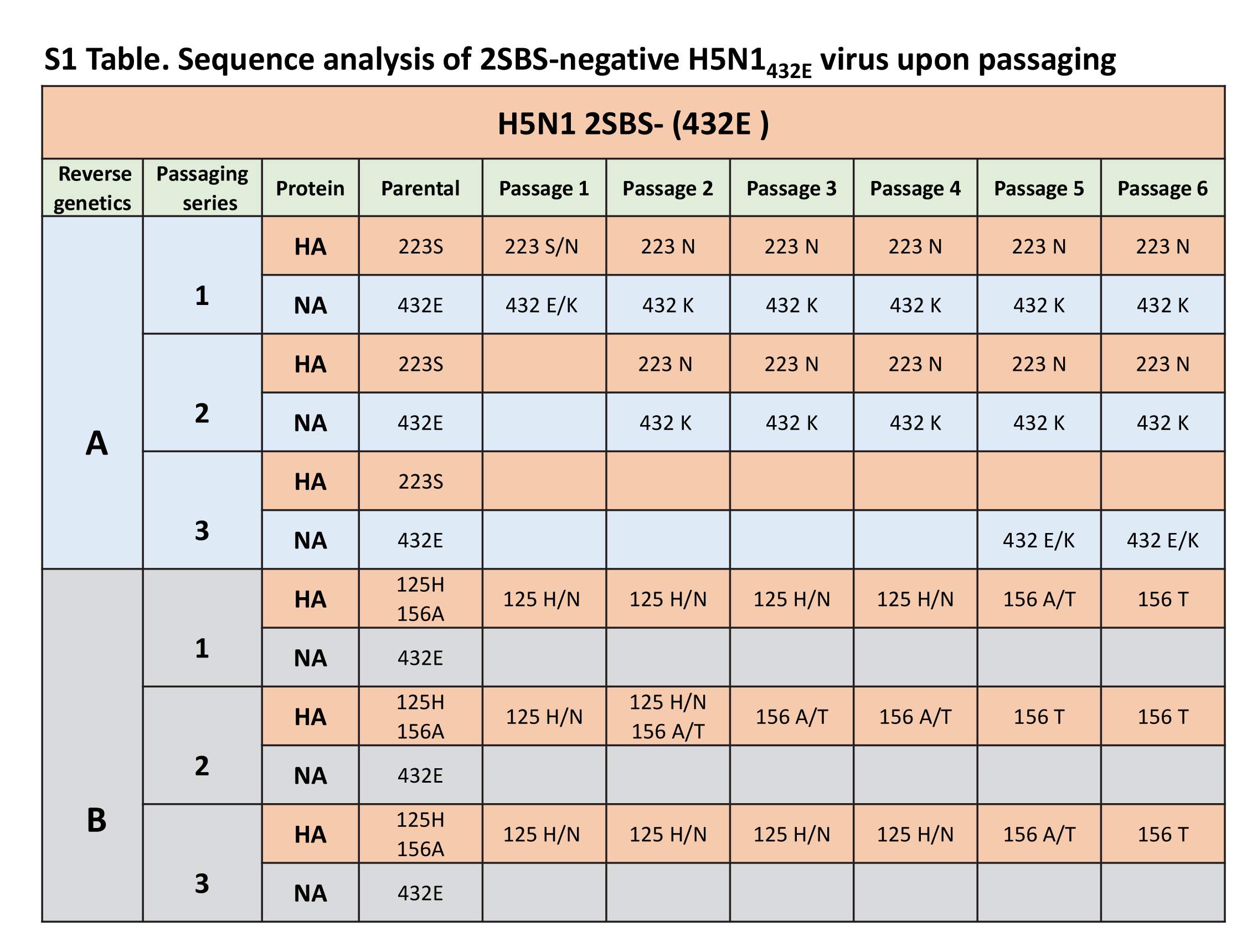

Supplement: S1 Table — (TIF) [file ppat.1008816.s007.tif]

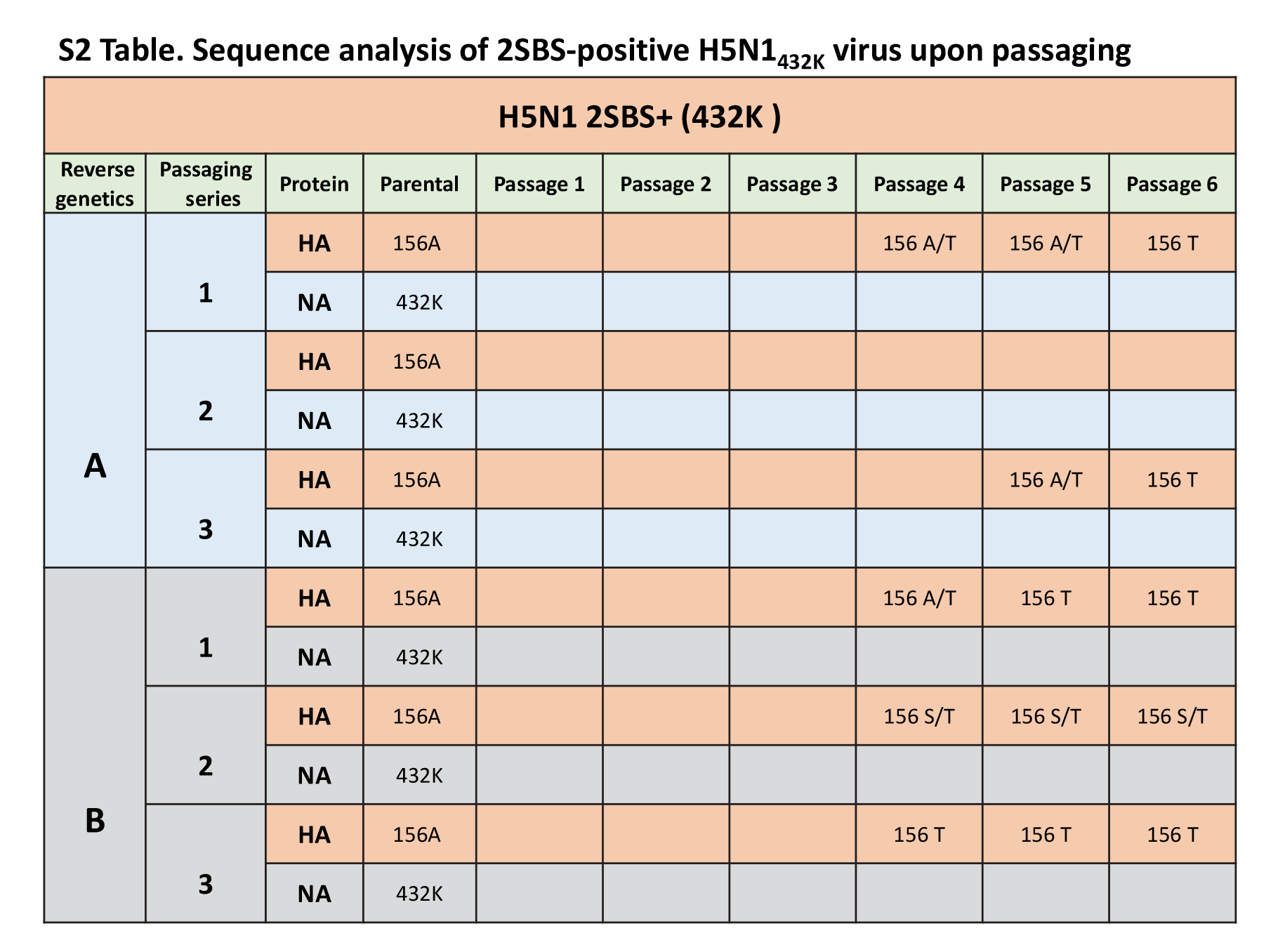

Supplement: S2 Table — (TIF) [file ppat.1008816.s008.tif]

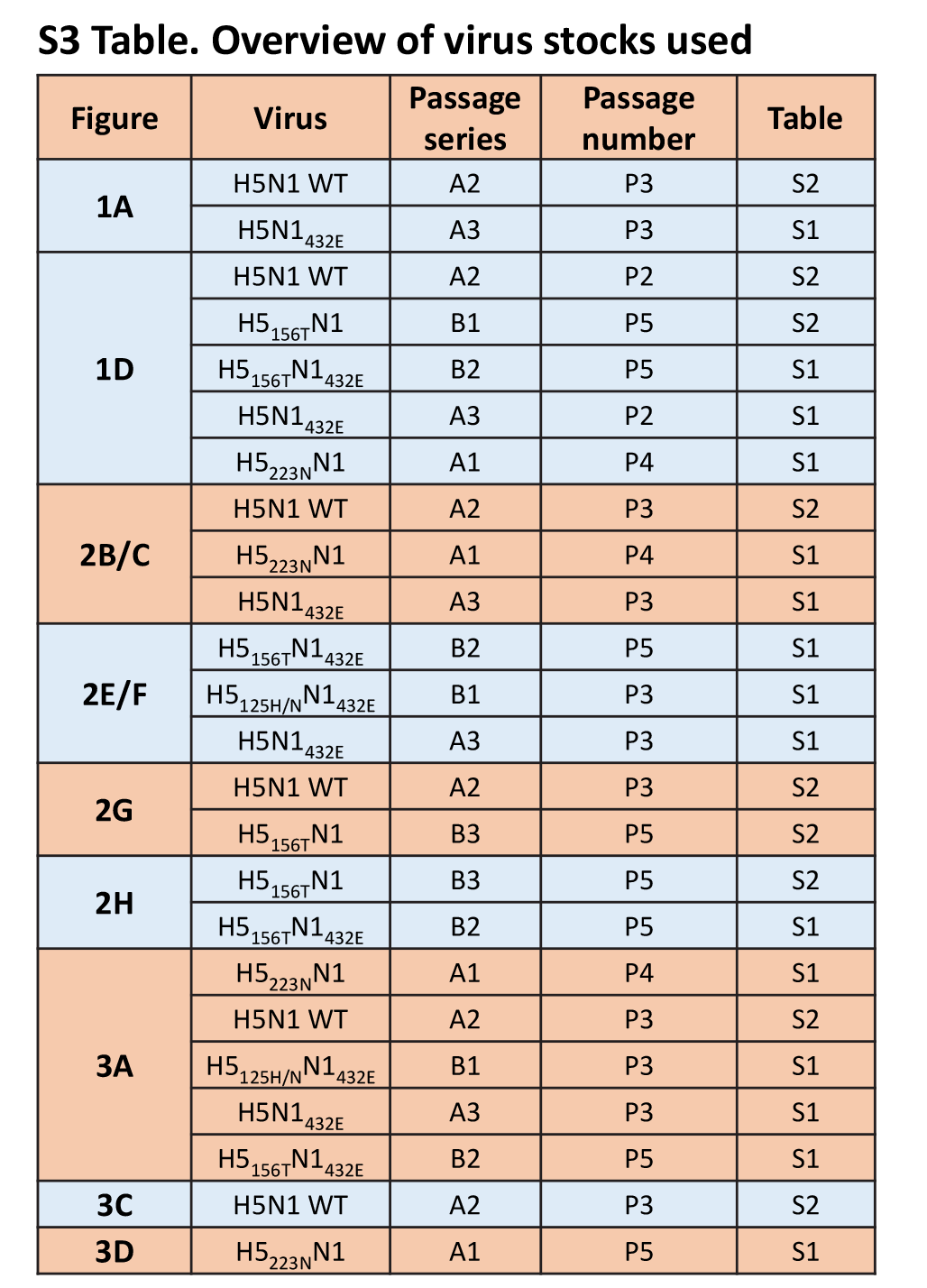

Supplement: S3 Table — (TIF) [file ppat.1008816.s009.tif]
